# Influence of filtering on the effective concentration and sterility of a 2% cyclosporine ophthalmic solution: a quality improvement perspective

**DOI:** 10.1186/s40780-023-00323-9

**Published:** 2023-12-27

**Authors:** Masakazu Ozaki, Toshihiko Kobayashi, Aki Fujinaga, Mitsuaki Nishioka, Kyoko Shikichi, Satoshi Okano, Yasuhito Sakai, Sayumi Fujii, Nobuaki Matsui, Miwako Takasago, Naoto Okada, Takahiro Yamasaki, Takashi Kitahara

**Affiliations:** 1 Pharmacy Department , Yamaguchi University Hospital, 1-1-1, Minami-kogushi, 755-8505 Ube, Yamaguchi Japan; 2 Division of Laboratory, Yamaguchi University Hospital, 1-1-1, Minami-kogushi, 755-8505 Ube, Yamaguchi Japan; 3 Department of Pharmacy, Fukuyama City Hospital, 5-23-1, Zao-cho, 721- 8511 Fukuyama, Hiroshima Japan; 4 Department of Oncology and Laboratory Medicine, Graduate School of Medicine, Yamaguchi University, 1-1-1, Minami-kogushi, 755-8505 Ube, Yamaguchi Japan; 5 Department of Clinical Pharmacology, Yamaguchi University Graduate School of Medicine, 1- 1-1, Minami-kogushi, 755-8505 Ube, Yamaguchi Japan

**Keywords:** Cyclosporine A, Particle size, In-hospital preparation, Filter operation, Ophthalmic solution

## Abstract

**Background:**

Pharmaceutical companies do not sell formulations for all diseases; thus, healthcare workers have to treat some diseases by concocting in-hospital preparations. An example is the high-concentration 2% cyclosporine A (CyA) ophthalmic solution. Utilizing a filter in sterility operations is a general practice for concocting in-hospital preparations, as is the case for preparing a 2% CyA ophthalmic solution. However, whether filtering is appropriate concerning the active ingredient content and bacterial contamination according to the post-preparing quality control of a 2% CyA ophthalmic solution is yet to be verified.

**Methods:**

We conducted particle size, preparation concentration, and bacterial contamination studies to clarify aforementioned questions. First, we measured the particle size of CyA through a laser diffraction particle size distribution. Next, we measured the concentration after preparation with or without a 0.45-µm filter operation using an electrochemiluminescence immunoassay. Finally, bacterial contamination tests were conducted using an automated blood culture system to prepare a 2% CyA ophthalmic solution without a 0.45 μm filtering. Regarding the pore size of the filter in this study, it was set to 0.45 μm with reference to the book (the 6th edition) with recipes for the preparation of in-hospital preparations edited by the Japanese Society of Hospital Pharmacists.

**Results:**

CyA had various particle sizes; approximately 30% of the total particles exceeded 0.45 μm. The mean ± standard deviation of filtered and non-filtered CyA concentrations in ophthalmic solutions were 346.51 ± 170.76 and 499.74 ± 76.95ng/mL, respectively (*p* = 0.011). Regarding bacterial contamination tests, aerobes and anaerobes microorganisms were not detected in 14 days of culture.

**Conclusions:**

Due to the results of this study, the concentration of CyA may be reduced by using a 0.45-µm filter during the preparation of CyA ophthalmic solutions, and furthermore that the use of a 0.45-µm filter may not contribute to sterility when preparing CyA ophthalmic solutions.

## Background

Research on drug discovery and advances in the pharmaceutical industry have resulted in the development of various drugs to support advanced medical care. Even with these advances, there is a lack of enough drugs for all known diseases. Therefore, in-hospital preparations are still being employed to treat various diseases. While concocting in-hospital preparations, filtration using filters is a general sterility practice [1]. International pharmacopeia, such as the European Pharmacopeia, the United States Pharmacopeia Convention and the Japanese Pharmacopeia, referenced in more than 130 countries worldwide, recommends using filters with a pore size of less than 0.45 μm to prepare in-hospital concocts [2].

A 2% cyclosporine A (CyA) ophthalmic solution effectively suppresses the immune responses in eye diseases and is a highly concentrated solution prepared in several countries [1–6]. CyA ophthalmic solutions are often prepared as oral or injectable formulations according to their concentration-related alcohol content [1–3]. Thus, when preparing a 2% CyA ophthalmic solution, an oral formulation tends to be selected [3, 5]. Topical administration of CyA is superior to systemic administration in terms of safety and adverse effects; furthermore, the 2% CyA ophthalmic solution is highly stable after preparation [7, 8]. However, the 2% CyA ophthalmic solution has not yet been successfully commercialized; therefore, the 2% CyA ophthalmic solution is prepared in-hospital worldwide [2]. The 2% CyA ophthalmic solution is also prepared in Japan, and the method of preparation is based on a book with recipes for the preparation of in-hospital preparations edited by the Japanese Society of Hospital Pharmacists (the 6th edition) [9]. The use of a filter with a pore size of 0.45 μm during the preparation of the 2% CyA ophthalmic solution is described in the book; however, the use of 0.45-µm filters when preparing the 2% CyA ophthalmic solution remains a concern.

CyA is an amorphous compound [10, 11], and the particle size is not always evenly distributed. Furthermore, the CyA oral solution used to prepare the 2% CyA ophthalmic solution is an emulsion [3, 12]. Generally, the particle size distribution of emulsions ranges from 0.1 to 10 μm. The diameter of CyA ranges from a few nanometers to tens of micrometers, and the particle size of CyA is variable [13]. Most studies on the particle size of CyA assessed using various methods have reported it being > 0.45 μm [13–16].

Moreover, the particle size of CyA decreases with a decrease in its concentration when diluted below 0.005%, but at a concentration of 0.05%, the particle size exceeds 0.45 μm [13, 16]. In contrast, in a study on the use of a CyA microemulsion formulation of concentration 0.05%, the particle size was smaller than 100 nm, depending on the particle size measurement method [17]. In addition, several studies have reduced the particle size of CyA by using various formulation techniques [13, 18, 19]. The oily nature of the CyA emulsion has long been recognized as a drawback; thus, studies of an aqueous and clear formulation of CyA have been conducted [19]. However, new formulations of CyA ophthalmic solution are yet to be prescribed, and this situation would persist even if a high-concentration 2% CyA ophthalmic solution is commercialized.

For high-concentration 2% CyA ophthalmic solutions to be used worldwide, there is no choice but to rely on in-hospital preparations using CyA oral solution. Filtration with a syringe filter when preparing in-hospital preparations is a standard operation for an aseptic procedure; however, the vulnerability of ophthalmic compounds and solutions to drug loss during filtration must be considered [20]. Moreover, regarding CyA, it is yet to be verified whether filtration is appropriate for post-preparation quality control considering active ingredient content and bacterial contamination. Therefore, we conducted this study to clarify whether using a 0.45-µm filter is appropriate for preparing a 2% CyA ophthalmic solution from the perspective of concentration and bacterial contamination after preparation.

## Methods

### Materials

To prepare the 2% CyA ophthalmic solution, Sandimmun^®^ Oral Solution 10% (Novartis, Switzerland) has been used [7, 8]. Therefore, we purchased Sandimmun^®^ Oral Solution 10% from Novartis Pharma K.K. (Japan). Olive oil was purchased from KENEI Pharmaceutical Co., Ltd. (Japan) because pure olive oil is often used for diluting Sandimmun^®^ Oral Solution from 10 to 2% [3]. The purchased olive oil was sterilized using dry-heat sterilization. For filtering the prepared 2% CyA ophthalmic solution, we used the 0.45-µm membrane filter (Millex^®^-HV Syringe Filter Unit, 0.45 μm, PVDF, 33 mm, gamma sterilized; Merck Millipore Ltd., Ireland). To measure the concentration of CyA, Ecrusis Cyclosporine was purchased from Roche Diagnostics K.K. (Japan). In addition, the 2% CyA ophthalmic solution has to be diluted to measure the concentration of CyA; ethanol purchased from KENEI Pharmaceutical Co., Ltd. (Japan) and Milli-Q water (Direct-Q^®^ UV 5; Merck KGaA, Germany) were used as diluent solvents. To perform the sterility test for bacterial contamination of the 2% CyA ophthalmic solutions prepared without the 0.45-µm filter operation, we purchased BD Bactek™ 23 F aerobic resin bottle and BD Bactek™ 22 F anaerobic resin bottle (Nippon Becton Dickinson Company, Ltd., Japan), which can confirm the presence of aerobic and anaerobic bacteria.

### Preparation of 2% CyA ophthalmic solutions with and without the 0.45-µm filter operation

**Fig. 1:**
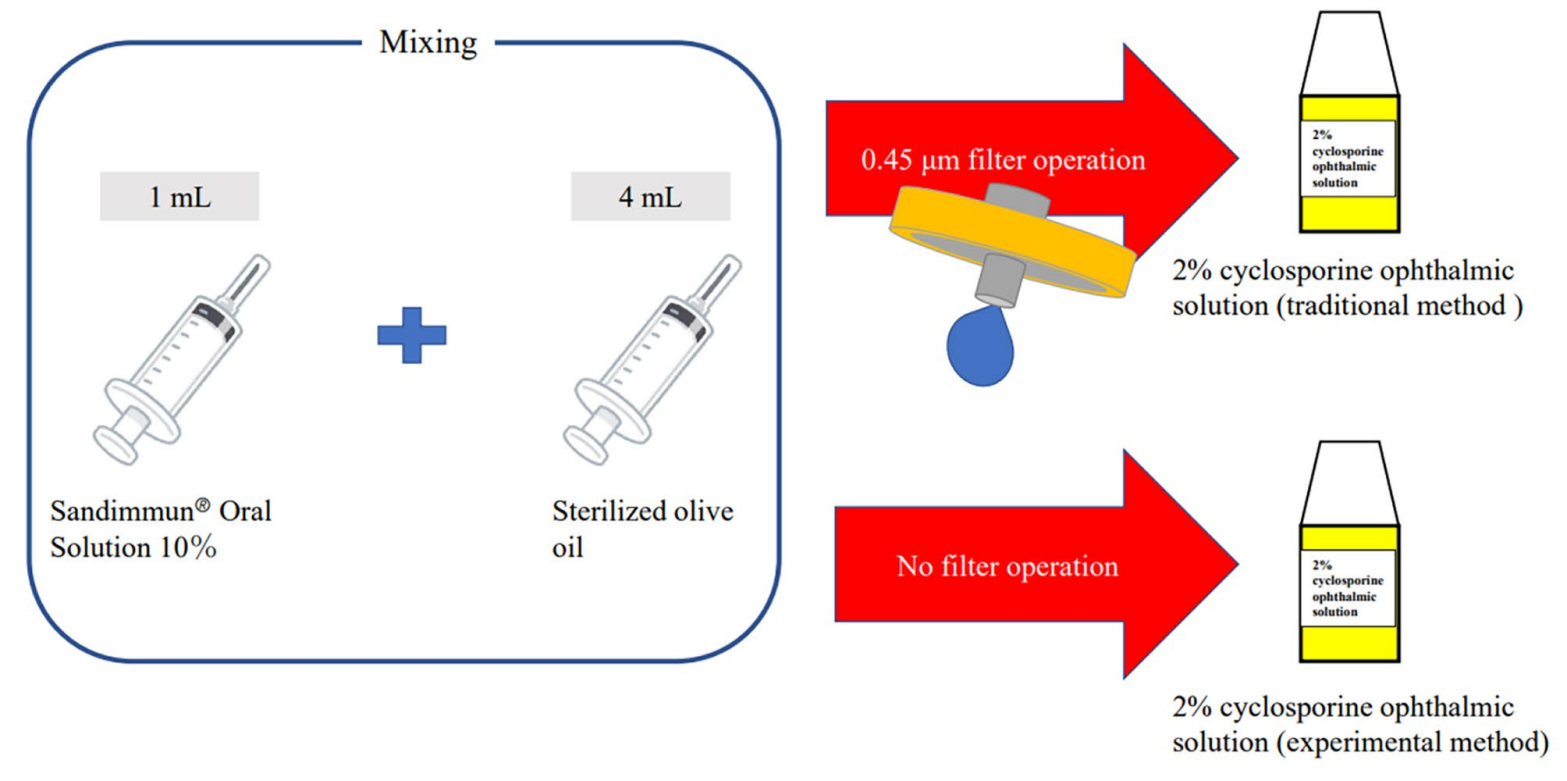
Method used for preparing a 2% cyclosporine A (CyA) ophthalmic solution with or without filtering through a 0.45-µm membrane

The 2% CyA ophthalmic solutions were prepared by precisely mixing 1 mL (specific gravity conversion: 0.930 g) of Sandimmun^®^ Oral Solution 10% and 4 mL (specific gravity conversion: 3.644 g) of sterilized olive oil. An electronic balance was utilized to confirm the weight during each weighing, and only those with a relative error of less than 1% were used. Subsequently, when 2% CyA ophthalmic solutions were filled into eyedrop containers, one was filtered using a 0.45-µm membrane filter, whereas the other was filled into the container without filtering (Fig. 1). Five individuals performed these operations. Each prepared both solutions with and without filtering, and 20 samples were prepared (two samples, with and without filtering, per preparer).

### Laser diffraction particle size distribution

A laser diffraction particle size distribution measuring apparatus (SZ-100; HORIBA Ltd., Japan) was used to measure particle sizes from 0.3 nm to 8 μm. Although it was preferable to measure the particle size of 2% CyA solutions from the perspective of this study, if the concentration of the target substance was too low, the measurement may become unstable. Therefore, in this study, 10% CyA solutions were measured. Sandimmun^®^ Oral Solution 10% was placed in a quartz cell 10 times and measured. The refractive index of CyA was set to 1.65 [14, 21, 22] and that of the solvent was set to 1.47 based on the information sheet of Sandimmun^®^ Oral Solution 10% (Novartis Pharma K.K., revised July 2022 (18th edition)). The viscosity of the dispersion medium was also calculated from the kinematic viscosity of the formulation and specific gravity of the dispersion medium; it was set to 45.264 mPa・s.

### Concentration measurement depending on the 0.45-µm filter operation

In instrumental analysis, automation of an analytical process can avoid the influence of operator technique and provide more reproducible results. Therefore, to measure the effects of filtrations using 0.45-µm on the concentration of CyA, the Cobas^®^ 8000 E602 module (Roche Diagnostics K.K., Japan), a reliable commercially available automated analyzer for electrochemiluminescence immunoassay (ECLIA), was used with Ecrusis Cyclosporine. The concentration range of CyA that can be measured using Ecrusis Cyclosporine is 30–2,000 ng/mL; however, it is necessary to dilute the actual solution to be measured as > 500 ng/mL from the reagent’s characteristics. As CyA is highly soluble in ethanol, the prepared 2% CyA ophthalmic solutions (20 mg/mL) were diluted 100-fold, and then another 100-fold with ethanol using an electronic balance and micropipette, resulting in a 10,000-fold (2,000 ng/mL) dilution. In addition, the procedure with the Cobas^®^ 8000 E602 module involves heating twice at 37 °C for 9 min; thus, the samples were further diluted 4-fold (500 ng/mL) with Milli-Q water to prevent ethanol volatilization. Regarding 25% ethanol volatility, a bottle containing 25% ethanol was stored twice with the lid opened in a constant temperature bath at 37 °C for 9 min, and no substantial changes in weight using an electronic balance were confirmed. Samples were prepared by five preparers, each preparing four samples (two samples, each with the filter and without the filter per preparer). When 25% ethanol was measured as a blank with the Cobas^®^ 8000 E602 module, a false positive value was obtained; thus, the actual measurement results were adjusted by subtracting the false positive value. The measurement results were subjected to a paired *t*-test, and the significance level was set to 0.05. Statistical analysis was performed using JMP^®^Pro16 software.

### Test for bacterial contamination of solutions prepared with and without the 0.45-µm filter operation

First, a positive control test of 2% CyA ophthalmic solution prepared with a 0.45-µm filter in a clean room was performed to determine whether the solution would be subject to contamination with the addition of *Staphylococcus aureus* ATCC^®^ 29,213™. The culture was regulated to 1.0 McFarland, equal to 3.0 × 10^8^ colony-forming unit (CFU)/mL. 1 mL of regulated *S. aureus* ATCC^®^ 29,213™ and 5 mL of the prepared 2% CyA ophthalmic solution were injected into a BD Bactek™ 23 F aerobic resin bottle and BD Bactek™ 22 F anaerobic resin bottle, respectively, and incubated in the BD BACTEC™ FX System (Nippon Becton Dickinson Company, Ltd., Japan). Positive results were obtained at 78 h 33 min and 9 h 48 min in the aerobic and anaerobic bottles, respectively, indicating that *S. aureus* can grow in the 2% CyA ophthalmic solution. Next, five preparers wearing surgical gloves and gowns prepared 10 unfiltered samples of 2% CyA ophthalmic solution in a clean room (two samples per preparer), and their bacterial culture tests were performed. The tests were performed for 14 days using the BD BACTEC™ FX System. For bacterial culture tests using the BD BACTEC™ FX System, the culture period is generally set to a maximum of 7 days. In this study, in order to avoid false negative results for the slow-growing HACEK group (Haemophilus, Aggregatibacter, Cardiobacterium, Eikenella, and Kingella species) that could be detected when cultured for more than 7 days [23], the culture period was doubled to 14 days. Each aerobic and anaerobic resin bottle was injected with 5 mL of sample. Subsequently, if the BD BACTEC™ FX System showed a positive result for the presence of bacteria in the resin bottle, we decided to identify the bacteria.

## Results

### Particle size distribution of CyA

**Fig. 2:**
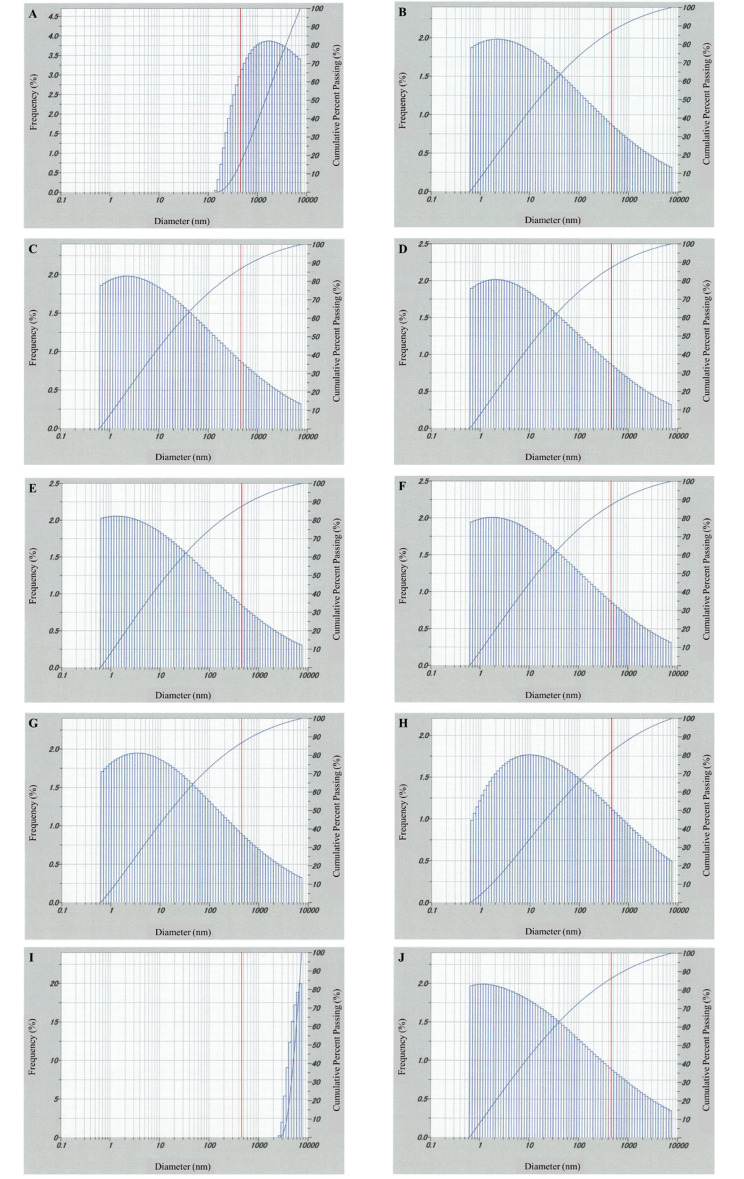
Results of 10 measurements obtained using the laser diffraction particle size distribution measuring device (The vertical red line indicates 450 nm)

The results of 10 measurements of Sandimmun^®^ Oral Solution 10% using the laser diffraction particle size distribution device are shown in Fig. 2. The cumulative percent passing at 450 nm was 15.987 (A), 87.088 (B), 86.912 (C), 87.146 (D), 87.593 (E), 87.211 (F), 86.886 (G), 81.965 (H), 0.000 (I), and 86.305% (J). The mean value was 70.7093%.

### Concentration of CyA in the 2% CyA ophthalmic solutions depending on whether the 0.45-µm filter operation was performed or not

The measurement results of 20 samples are shown in Table 1. The mean ± standard deviation of CyA concentration in the ophthalmic solutions prepared with a 0.45-µm filter was 346.51 ± 170.76 ng/mL. In contrast, the mean ± standard deviation of CyA concentration in the ophthalmic solutions prepared without the filter was 499.74 ± 76.95 ng/mL. A significant difference in the mean concentration of CyA was observed between the solutions, with a *p*-value of 0.011. The coefficients of variation results of the solutions were 0.493 and 0.154, respectively.

**Table 1:** Measurement results of the concentration of cyclosporine A in 2% cyclosporine ophthalmic solutions

Preparer	Filtered sample No.	Concentration (ng/mL)	Unfiltered sample No.	Concentration (ng/mL)	*p*-value*
A	1	501.83	1	546.63	0.011
	2	429.03	2	525.77	
B	3	398.43	3	380.97	
	4	374.00	4	465.23	
C	5	338.30	5	577.30	
	6	641.33	6	644.13	
D	7	84.40	7	466.67	
	8	85.13	8	509.90	
E	9	326.43	9	441.57	
	10	286.23	10	439.20	
	Mean ± S.D.	346.51 ± 170.76	Mean ± S.D.	499.74 ± 76.95	
	C.V.	0.493	C.V.	0.154	

* paired *t*-test

Abbreviations: S.D., standard deviation; C.V., coefficient of variation

### Sterility of the 2% CyA ophthalmic solutions prepared by five preparers without the 0.45-µm filter operation

The detection results of the positive control and 10 samples are shown in Table 2. In these 10 samples, aerobic and anaerobic microorganisms were not detected after 14-day culturing in the BD BACTEC™ FX System. Therefore, no tests were performed to identify bacteria.

**Table 2:** Detection results of aerobic and anaerobic microorganisms in 2% cyclosporine ophthalmic solutions

Preparer	Detection time of aerobic microorganisms (hours:minutes)	Detection time of anaerobic microorganisms (hours:minutes)
Positive control	78:33	9:48
A	Not detected	Not detected
B	Not detected	Not detected
C	Not detected	Not detected
D	Not detected	Not detected
E	Not detected	Not detected

Note: Positive control test was performed with the addition of *Staphylococcus aureus* ATCC^®^ 29,213™, regulated to 1.0 McFarland, equal to 3.0 × 10^8^ colony-forming unit (CFU)/mL. 1 mL of regulated S. aureus ATCC^®^ 29,213™ and 5 mL of the prepared 2% CyA ophthalmic solution were mixed; Bacterial culture tests were performed for up to 336 h; 5 mL of 2% cyclosporine ophthalmic solution was injected into each resin bottle

## Discussion

The results of the CyA particle size distribution analysis showed that the formulation contained approximately 30% CyA with a particle size of > 450 nm. To our knowledge, no previous study has reported the percentage of CyA particles with a particle size larger than 450 nm. In this study, 10% CyA solutions were used as a substitute for 2% CyA solutions in consideration of measurement stability; particle size distributions determined in our study were similar to those in previous studies on 0.05% CyA [13, 16]. Therefore, unless the concentration is extremely low, such as below 0.005% [16], particle size distribution is expected to be similar between 10% and 2% solutions.

As of results (A) and (I), the particle sizes are unstable and large compared to other results; this is considered to be peculiar to amorphous materials [24, 25]. Amorphous materials reportedly crystallize at temperatures considerably below the glass transition temperature at which they crystallize; on the contrary, amorphous materials also exhibit local mobility, which causes aggregation [24]. It has been reported that the average size of amorphous CyA particles crystallized in water is approximately 60 μm [25], and the particle size distributions of (A) and (I) were considerably smaller than that. Aggregation size is generally smaller than crystallization size; therefore, the results of (A) and (I) could be attributed to aggregation rather than crystallization. Moreover, in past study, the particle size of Sandimmun^®^ formulation dispersed in water was measured, and it was reported that the mean particle size was approximately 1400 nm [26]. This finding is likely because of aggregation, which could also be attributed to results (A) and (I), although the cause of occurrence of aggregation could not be determined in this study. Thus, a filter smaller than 450 nm in pore size might not be appropriate to prepare CyA formulations, including the 2% CyA ophthalmic solution. On the contrary, CyA microemulsion formulations are designed to be small particle size [26], and selecting microemulsion formulations for the preparation of CyA ophthalmic solutions might minimize particle size issues. Further study using microemulsion formulations is necessary in the future.

Regarding the concentration of CyA in 2% CyA ophthalmic solution, the filtered solution showed a decrease in CyA concentration by approximately 30% compared to the unfiltered solution, which was almost consistent with the results of particle size distribution. Furthermore, the difference in the coefficients of variation indicates that CyA does not have a constant particle size, and the degree to which it is filtered varies with different preparations. All CyA molecules would theoretically pass through in the absence of a filter operation; thus, the overall mean concentration should be normally distributed with low variance. In contrast, when a filter is used, the passage of 0.45 μm can cause unstable results, such as results (A) and (I) of particle size distribution measurements (Fig. 2). This result shows that CyA with different particle sizes was considerably affected by the filter. Especially, for filtered samples 7 and 8 of preparer D (Table 1), the influence of the filter operation was noticeable. This finding may be related to aggregation, causing the particle size to increase; consequently, the particles could not have passed through the filter. However, it is not clear why this possible aggregation phenomenon occurred only in the operation of preparer D. Contrarily, the average value of CyA in the unfiltered 2% CyA ophthalmic solution should be 500 ng/mL, which was confirmed by the measurement in the Cobas^®^ 8000 E602 module.

In this study, ECLIA, a recently developed measurement method, was utilized to measure the CyA concentration in CyA ophthalmic solutions. ECLIA is a concise and reliable measurement method compared to high-performance liquid chromatography (HPLC). However, ECLIA often requires sample dilutions in clinical practice; dilutions were also employed in this study. Therefore, attention should be paid to the solubility and volatility of the solvent. In this study, CyA was diluted in ethanol; CyA has a high solubility in ethanol, and 25% ethanol was not substantially volatilized.

Based on the results of sterility testing of 2% CyA ophthalmic solutions prepared without using a 0.45-µm filter, if aseptic operations are performed in a clean room, the use of 0.45-µm filters does not contribute to sterility. According to previous studies, to achieve sterility, filters of pore size 0.22 μm or smaller are required [27]. Furthermore, in a recent study, some special ultramicro bacteria or filterable bacteria, or both, have been found to pass through filters of pore size 0.22 μm [28]. Considering these findings, the use of 0.45-µm filters cannot be considered a sterile approach to avoid bacterial contamination. To achieve sterility of the formulation, an aseptic operation in a clean room is essential.

The strength of this study is that it successfully clarifies the previously overlooked issue of filter incompatibility when preparing CyA ophthalmic solutions. Nevertheless, the study has some limitations. In this study, a clinical evaluation was not performed; therefore, the difference in clinical effect due to the influence of filter operation could not be clarified. However, it has been reported that a mere 0.05% concentration difference of CyA ophthalmic solution affected the CyA blood concentration [3]. Moreover, in CyA ophthalmic formulations, 2% is defined as high concentration; therefore, although it is difficult to ascertain to what extent the decrease in CyA concentration, as observed in this study, affects pharmacological effects, we believe that the decrease of CyA concentration in this study has critical implications. Further research is necessary to determine the need for filter operation through clinical evaluation with and without a 0.45 μm filter.

## Conclusions

To concoct in-hospital preparations, attention must be paid to effective concentration and sterility. In addition, considering the sterility and effective concentration of CyA preparations, the use of a 0.45-µm filter may not be a rational method for preparing a 2% CyA ophthalmic solution. This kind of perspective by pharmacists who are pharmaceutical experts is extremely important for the quality of medical care.

## Data Availability

All data generated or analysed during this study are included in this published article.

## Abbreviations

CyA: cyclosporine A
ECLIA: electrochemiluminescence immunoassay
HPLC: high-performance liquid chromatography
